# B Cell Repopulation After Alemtuzumab Induction—Transient Increase in Transitional B Cells and Long-Term Dominance of Naïve B Cells

**DOI:** 10.1111/j.1600-6143.2012.04012.x

**Published:** 2012-07

**Authors:** S Heidt, J Hester, S Shankar, P J Friend, K J Wood

**Affiliations:** a Transplant Research Immunology Group, Nuffield Department of Surgical Sciences, University of OxfordOxford, UK; a Oxford Transplant Centre, Nuffield Department of Surgical Sciences, University of OxfordOxford, UK

**Keywords:** Campath-1H, conversion, depletion, renal transplant, regulatory B cells

## Abstract

In organ transplantation, the composition of the B-cell compartment is increasingly identified as an important determinant for graft outcome. Whereas naïve and transitional B cells have been associated with long-term allograft survival and operational tolerance, memory B cells have been linked to decreased allograft survival. Alemtuzumab induction therapy effectively depletes B cells, but is followed by rapid repopulation up to levels exceeding base line. The characteristics of the repopulating B cells are currently unknown. We studied the phenotypic and functional characteristics of B cells longitudinally in 19 kidney transplant recipients, before and at 6, 9 and 12 months after alemtuzumab induction therapy. A transient increase in transitional B cells and cells with phenotypic characteristics of regulatory B cells, as well as a long-term dominance in naïve B cells was found in alemtuzumab-treated kidney transplant recipients, which was not influenced by conversion from tacrolimus to sirolimus. At all time-points after treatment, B cells showed unaltered proliferative and IgM-producing capacity as compared to pretransplant samples, whereas the ability to produce IgG was inhibited long-term. In conclusion, induction therapy with alemtuzumab results in a long-term shift toward naïve B cells with altered phenotypic and functional characteristics.

## Introduction

Humoral immunity is increasingly recognized as an important component of the alloimmune response. It is clear that alloantibodies produced by B cells can cause (hyper)acute graft rejection and it has been postulated that production of donor specific alloantibodies after transplantation may be an important cause of late graft loss (1,2). Interestingly, and in contrast, novel data suggest that B cells may play an important role in allograft survival and the development of operational transplant tolerance (reviewed in Ref. 3).

Recently, a B-cell signature of tolerance has been described in immunosuppressive drug free long-term surviving kidney transplant recipients (KTRs), including an increase in the total number of peripheral B cells (4) and a relative increase in naïve and transitional B cells, when compared to KTRs with stable graft function or biopsy proven chronic rejection (5,6). A distinct, but also B-cell dominated signature of tolerance was identified in a separate cohort of long-term immunosuppression free KTRs (7). Taken together, these data suggest a potential role for B cells in the development and/or maintenance of operational tolerance in KTRs (8).

Characterization of the immune phenotype of patients with surviving allografts in the absence of immunosuppressive drugs compliments experimental studies exploring novel strategies to actively induce transplantation tolerance. In a nonhuman primate model of islet transplantation, long-term allograft survival achieved by T and B cell depletion with anti-thymocyte globulin (ATG) and rituximab was associated with the emergence and persistence of immature and transitional B cells (9). These data suggest that therapies that drive the B cell compartment towards a “tolerant” phenotype could potentially aid in the establishment of transplantation tolerance.

The anti-CD52 specific humanized monoclonal antibody alemtuzumab rapidly depletes T cells, B cells, NK cells and monocytes from the circulation, and is increasingly being used as induction therapy for kidney transplantation (10). After depletion, monocytes rapidly repopulate, followed by NK cells and B cells (11–13). Strikingly, although T cells can take years to repopulate to pretreatment levels, B cells often repopulate up to levels exceeding pre-treatment levels within a year after treatment (12,14).

Although repopulating B cells of patients with multiple sclerosis (MS) treated with alemtuzumab have been shown to be mainly naïve (14), information about the composition of the repopulating B cell pool and the influence of maintenance immunosuppressive therapy in KTRs after alemtuzumab induction is scarce (15). In the light of the increasingly appreciated role of B cells in transplant rejection and tolerance, characterization of repopulating B cells after alemtuzumab induction is of great interest. We therefore hypothesized that repopulating B cells in alemtuzumab treated KTRs would show an altered phenotypic and functional profile compared to that pretransplant and that this would not be impacted by posttransplant immunosuppression.

## Materials and methods

### Patients

KTRs treated with alemtuzumab induction therapy (two doses of 30 mg i.v.) were included (n = 19). Fifteen patients received maintenance immunosuppressive therapy consisting of tacrolimus (target trough level 5–8 ng/mL), MMF (500 mg, twice daily) and steroids. Four patients did not receive steroids and were converted from tacrolimus to low dose sirolimus (adjusted to 5–8 ng/mL) at 6 months, followed by MMF withdrawal at 12 months. The study was approved by Oxfordshire Research Ethics Committee B under the reference numbers 07/H0603/42 and C02.225. Patients were recruited after informed consent and blood was taken before and at several time points up to 12 months after transplantation.

### Cells

Peripheral blood mononuclear cells (PBMC) were isolated by Ficoll-Paque (GE Healthcare, Uppsala, Sweden) gradient centrifugation and stored in liquid nitrogen until further use. B cells were immunomagnetically isolated using Dynabeads CD19 pan B and Detach-a-Bead CD19 (Invitrogen, San Diego, CA, USA). Cell cultures were performed in culture medium consisting of Iscove's modified Dulbecco's medium (IMDM) supplemented with 10% fetal calf serum (FCS), 100 U/mL penicillin, 100 μg/mL streptomycin (all from PAA Laboratories, Pasching, Austria), 0.05 mM 2-mercaptoethanol (Sigma-Aldrich, St. Louis, MO, USA) and ITS (insulin 5 μg/mL, transferrin 5 μg/mL and selenium 5 ng/ml; Sigma-Aldrich).

### Flow cytometry

Flow cytometric analysis (FCM) was performed according to standard protocols using the following antibodies (clone): CD19 (SJ25C1), CD27 (M-T271), CD24 (ML5), CD5 (UCHT2), IgD (IA6–2), IgM (G20–127) (all from BD Biosciences, Oxford, UK), CD10 (ALB1) (Beckman Coulter, Fullerton, CA, USA), CD20 (2H7), CD38 (HIT2) (eBioscience, San Diego, CA, USA), CD1d (51.1) (Biolegend San Diego, CA, USA) or relevant isotype controls.

### B-cell activation

B cells were cultured at 1 × 10^5^ cells/well in 96-well roundbottom plates (Corning, Amsterdam, the Netherlands) and activated with 500 ng/mL of agonistic anti-CD40, 25 ng/mL of interleukin (IL)-10 (both from R&D systems, Abingdon, UK), 100 ng/mL of IL-21 (Invitrogen), 100 U/mL of IL-2 (Chiron, Emeryville, CA, USA) and 2.5 μg/mL of ODN-2006 CpG (Hycult Biotechnology, Uden, the Netherlands).

### Proliferation assay

B cells were activated as described earlier for 7 days. At day 6, supernatants were collected for Ig detection and 1 μCi ^3^H-TdR (Perkin Elmer, Cambridge, UK) was added per well for the last 16 h of culture. ^3^H-TdR incorporation was measured using a liquid scintillation counter (Wallac, Turku, Finland).

### Immunoglobulin production

ELISA and ELISPOT assays to quantify IgM and IgG levels in culture supernatants and the number of B cells producing IgM and IgG were performed as described previously (16).

### Statistics

The repeated measures ANOVA with posttesting by Dunnett Multiple Comparisons Test was used for comparisons of variables in time, whereas the unpaired T test was used to analyze differences between treatment groups; p values < 0.05 were considered significant. Results in the text are expressed as mean ± SD.

## Results

### Repopulating B cells after alemtuzumab induction show a naïve phenotype

**Figure 1:**
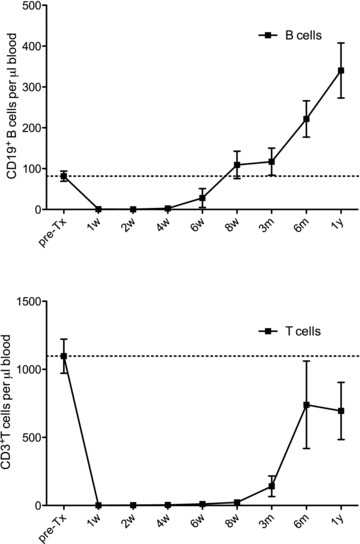
(A) Absolute number of CD19^+^ B cells and (B) CD3^+^ T cells in peripheral blood of alemtuzumab treated KTRs up to 12 months after treatment (n = 10) The absolute number of cells was determined by multiplying the respective percentages obtained by flow cytometry by absolute lymphocyte counts from clinical laboratory reports.The dotted lines represent pretransplant values.

After alemtuzumab-induced leukocyte depletion, B cells repopulated the peripheral blood of KTRs from 6 weeks onward and exceeded base line levels from 6 months (Figure 1). T cells started to repopulate after 3 months, not reaching base line levels within the 1-year time frame, confirming previous reports (11,12).

**Figure 2:**
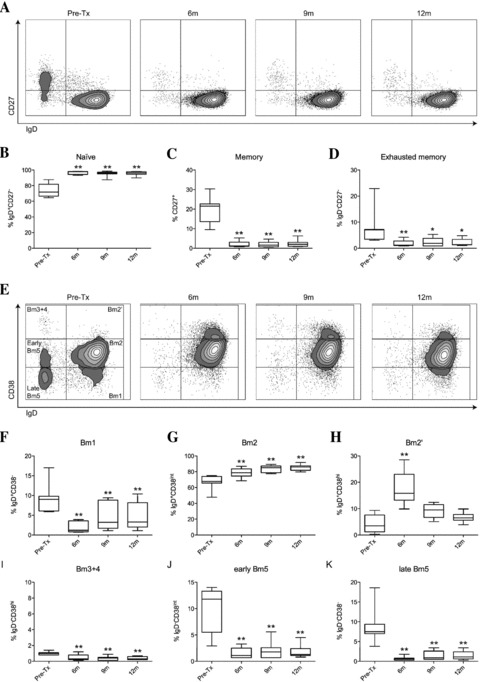
Phenotype of repopulating B cells after alemtuzumab induction therapy up to 12 months after transplantation (A) Representative dot plots of repopulating B cells using the CD27-IgD classification. (B) Percentage of naïve B cells (IgD^+^CD27^−^) within CD19 gate, (C) percentage of memory B cells (both IgD^−^CD27^+^ and IgD^+^CD27^+^) within CD19 gate and (D) percentage of exhausted memory B cells (IgD^−^CD27^−^) within CD19 gate (n = 7). (E) Representative dot plots of repopulating B cells using the Bm1-Bm5 classification. (F) Percentage of Bm1 cells (IgD^+^CD38^−^) within CD19 gate, (G) percentage of Bm2 cells (IgD^+^CD38^int^) within CD19 gate, and (H) percentage of Bm2’ cells (IgD^+^CD38^hi^) within CD19 gate, (I) percentage of Bm3 + 4 cells (IgD^−^CD38^hi^) within CD19 gate, (J) percentage of early Bm5 cells (IgD^−^CD38^int^) within CD19 gate and (K) percentage of late Bm5 (IgD^−^CD38^−^) within CD19 gate (n = 7). All statistics: repeated measures ANOVA with posttesting by Dunnett Multiple Comparisons Test, *p < 0.05, **p < 0.01.

B-cell differentiation stages can be identified by FCM using various classification schemes, of which the CD27-IgD (17) and the Bm1–Bm5 classification (18) are most commonly used. Using the CD27-IgD classification scheme (Figure 2A), we observed that following alemtuzumab treatment, there was a clear shift to lower levels of memory B cells in the peripheral blood; 19.1 ± 7.0% memory B cells (including IgD^+^CD27^+^ nonswitched memory B cells and IgD^−^CD27^+^ switched memory B cells) preinduction compared to 2.0 ± 1.6% at 6 months posttransplant (p < 0.01). The proportion of memory B cells remained low in alemtuzumab-treated KTRs for 12 months after treatment (2.4%± 1.9, p < 0.01, Figure 2B). The IgD^−^CD27^−^ B-cell population, which has been described as comprising exhausted memory B cells (19), was also decreased from 7.9 ± 6.9% preinduction to 1.9 ± 1.2% at 6 months (p < 0.01), remaining low up to 12 months (2.3 ± 1.4%, p < 0.05, Figure 2C). Consequently, the naive B-cell compartment (IgD^+^CD27^−^) was highly enriched from a mean of 73.6 ± 8.3% pretreatment to 96.7 ± 2.1% at 6 months after treatment (p < 0.01), and remained high at 12 months after transplantation (95.6 ± 2.8%, p < 0.01, Figure 2D).

When using IgD and CD38 to identify B cells into Bm1–Bm5 subsets (Figure 2E), we observed a decrease in Bm1 cells from 9.3 ± 3.8% pretreatment to 2.0 ± 1.4% at 6 months (p < 0.01) that remained attenuated up to 12 months (5.0 ± 3.5%, p < 0.01, Figure 2F). This was mainly due to the depletion of memory B cells that had not undergone class switching (non-switched memory B cells) that reside in this gate besides “virgin naïve” B cells, as determined by CD27 positivity (data not shown). Bm2 cells (comprising activated naïve B cells) were increased from 67.2 ± 9.2% before treatment to 78.7 ± 6.3% at 6 months (p < 0.01). This subpopulation continued to rise at 12 months after treatment, up to 85.3 ± 4.1% (p < 0.01, Figure 2G).

**Figure 3:**
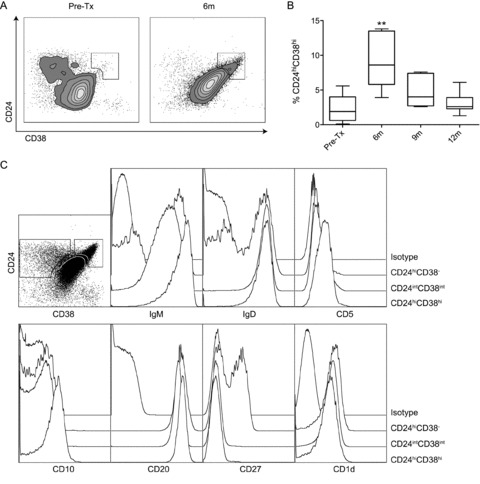
Transient increase of B cells with a Breg phenotype after alemtuzumab induction therapy (A) Representative dot plots of CD19^+^CD24^hi^CD38^hi^ B cells pretransplantation and at 6 months after transplantation. (B) Percentage of CD24^hi^CD38^hi^ B cells within CD19 gate (n = 7), statistics: repeated measures ANOVA with posttesting by Dunnett Multiple Comparisons Test, **p < 0.01. (C) CD24^hi^CD38^hi^ B cells have the phenotypic characteristics of Breg cells; IgM^hi^ and IgD^hi^, CD5^+^, CD10^+^, CD20^hi^, CD27^−^ and CD1d^+^.

Cells in the Bm2’ gate were transiently increased from 4.3 ± 3.5% pretreatment to 18.0 ± 6.7% at 6 months (p < 0.01), after which levels dropped near to baseline levels (6.7 ± 1.9%, p = ns, Figure 2H). Because these cells were mainly CD27^−^, they have the phenotypic characteristics of transitional B cells, rather than pregerminal B cells, which are CD27^+^ (20,21). Recently, a population of human regulatory B cells (Breg) residing within the transitional B-cell compartment expressing high levels of CD24 and CD38 has been described (22). In alemtuzumab-treated KTRs, we observed a transient increase in peripheral B cells expressing high levels of CD24 and CD38 at 6 months after transplantation (Figure 3A; 2.2 ± 2.0% pretreatment vs. 8.9 ± 3.9% at 6 months (p < 0.01)). These cells had the phenotypic characteristics of human Breg (22), because these cells were IgM^hi^IgD^hi^CD5^+^CD10^+^CD20^+^CD27^−^CD1d^+^ (Figure 3C). Analysis at later time points demonstrated that this subpopulation then declined to lower levels; 4.5 ± 2.1% at 9 months (p = ns) and 3.1 ± 1.5% at 12 months (p = ns, Figure 3B).

The germinal centre Bm3 + 4 subsets were virtually absent (Figure 2I), as has been described previously for peripheral blood (18). Consistent with results from the IgD-CD27 classification system, the percentage of both early and late memory cells (Bm5) were profoundly decreased after alemtuzumab induction. Early Bm5 cells decreased from 10.1 ± 4.3% to 1.6 ± 1.0% at 6 months (p < 0.01), remaining low at 12 months (1.9 ± 1.3%, p < 0.01, Figure 2J), whereas late Bm5 cells decreased from 8.9 ± 4.6% to 0.7 ± 0.5% at 6 months (p < 0.01), remaining low at 12 months (1.4 ± 1.1%, p < 0.01, Figure 2K).

For five KTRs, we had the opportunity to determine the absolute number of B cells in each subset in fresh blood samples pretransplant and at 6 months after transplantation. The observed changes in the absolute number of cells in each B-cell subset confirmed our findings analyzing stored frozen PBMC, indicating that in the peripheral blood of KTRs after alemtuzumab induction therapy the composition of B cells subsets is altered compared to that found pretransplant; naïve and transitional B cells are increased, whereas the absolute number of memory B cells is significantly decreased (Figure S1).

### Repopulating B cells have an altered response to polyclonal stimulation

**Figure 4:**
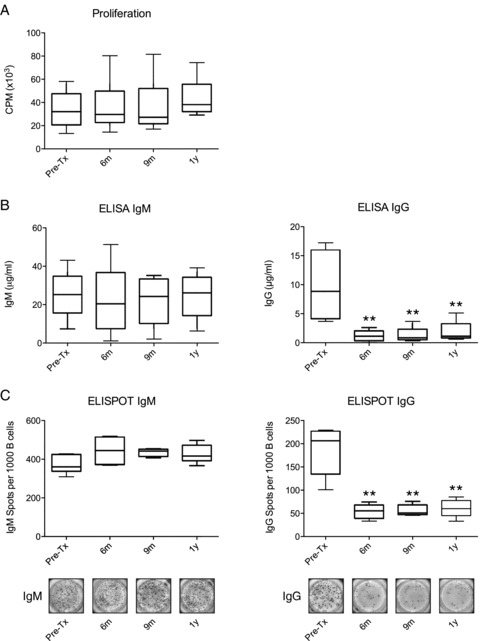
Functional analysis of repopulating B cells after alemtuzumab induction therapy (A) B-cell proliferation after polyclonal stimulation pretransplant and up to 12 months after alemtuzumab treatment analyzed by ^3^H-TdR incorporation (n = 6). (B) IgM and IgG concentration in supernatants from polyclonally activated B cells pretransplant and up to 12 months after alemtuzumab treatment analyzed by ELISA (n = 5). (C) Results from IgM and IgG ELISPOT of polyclonally activated B cells pretransplant and up to 12 months after alemtuzumab treatment (n = 6). All statistics: repeated measures ANOVA with posttesting by Dunnett Multiple Comparisons Test, **p < 0.01.

To investigate the functional properties of repopulating B cells from KTRs-treated with alemtuzumab induction therapy, B cells were stimulated polyclonally and found to proliferate with the same extent to that of B cells before alemtuzumab induction (Figure 4A, p = ns for any time point). However, whereas similar amounts of IgM were detected in the supernatant of the B-cell cultures both pre- and postinduction (p = ns for any time point), the amount of IgG detected was profoundly decreased from 9.8 ± 6.0 μg/mL in pretransplant B cell cultures to 1.1 ± 1.0 μg/mL at 6 months (p < 0.01), 1.3 ± 1.0 μg/mL at 9 months (p < 0.01) and 1.8 ± 1.0 μg/mL at 12 months after treatment (p < 0.01, Figure 4B).

To verify whether this was due to a reduction of the number of cells producing IgG, we performed Ig ELISPOT assays on polyclonally stimulated B cells. Compared to pretreatment cultures, the number of B cells producing IgM was unaltered (p = ns for any time point). In contrast, the number of B cells producing IgG after polyclonal stimulation was dramatically decreased from 186 ± 52 spot-forming cells (SFC) per 1000 B cells pretreatment to 54 ± 15 SFC at 6 months (p < 0.01), 56 ± 12 SFC at 9 months (p < 0.01) and 60 ± 19 SFC at 12 months posttreatment (p < 0.01, Figure 4C).

### Long-term immunosuppression does not influence B-cell repopulation

To establish whether maintenance immunosuppression would influence the distribution of B-cell subsets, we determined the phenotype and composition of B cells in the peripheral blood of alemtuzumab-treated KTRs who were enrolled in a conversion trial in which immunosuppression was converted from tacrolimus to low dose sirolimus at 6 months after transplantation. B-cell subsets at 12 months in these patients were compared to those in alemtuzumab-treated KTRs who remained on standard triple therapy. As shown in Table 1, we did not find any differences in B-cell subsets between patients converted to sirolimus and patients that remained on standard triple therapy.

**Table 1:** Comparison of B cells subsets in percentages ± SD at 12 months after transplantation between patients converted to sirolimus at 6 months and patients on standard triple therapy

	B cells	Naïve	Memory	DN	Bm1	Bm2	Bm2′	Bm3 + 4	Early Bm5	Late Bm5	Transitional
No conversion (n = 7)	24.6 ± 19.8	95.6 ± 2.8	2.4 ± 1.9	2.3 ± 1.4	5.0 ± 3.5	85.3 ± 4.1	6.7 ± 1.9	0.4 ± 0.2	1.9 ± 1.3	1.4 ± 1.1	3.1 ± 1.5
Conversion (n = 4)	26.6 ± 6.2	94.8 ± 3.9	2.9 ± 1.9	2.3 ± 2.1	3.8 ± 4.1	87.2 ± 8.2	4.7 ± 1.4	0.3 ± 0.1	1.9 ± 1.1	2.7 ± 2.9	3.0 ± 0.5
p-Value	0.85	0.69	0.65	0.97	0.60	0.60	0.11	0.48	0.98	0.32	0.84

Statistics: unpaired T test.

## Discussion

Increasing evidence suggests that B cells may contribute to transplant tolerance, potentially resulting in successful long-term graft outcome. In this study, we have shown that after alemtuzumab induction, the repopulating B-cell compartment is altered compared to that pretransplant, comprising mainly naïve B cells that produce IgM upon activation, and that these remain the dominant cell type for at least 12 months after alemtuzumab therapy. Furthermore, we have shown that following alemtuzumab treatment there are dynamic changes in the repopulating B-cell pool with a transient increase in transitional B cells, including B cells with phenotypic characteristics of Breg.

The composition of the B-cell compartment has been identified as an important factor for graft outcome. An increase in the number of naïve B cells and percentage of transitional B cells has been described in long-term immunosuppressive drug free tolerant KTRs, suggesting that a shift towards a naïve/transitional B-cell phenotype might be a prerequisite for the development of tolerance (6). Similarly, in a nonhuman primate model of islet transplantation, B-cell reconstitution after rituximab was dominated by immature and transitional B cells whose persistence was associated with long-term insulin independence (9). Moreover, in KTRs treated with rituximab for CD20^+^ acute rejection, a decrease in naïve B cells was associated with graft loss (23).

After alemtuzumab induction, we observed a shift toward naïve B cells in peripheral blood, as determined by different B-cell classification schemes. Consequently, B cells with a memory phenotype were virtually absent for at least 12 months after induction therapy. We also showed that alemtuzumab treatment resulted in a transient increase in cells that have phenotypic characteristics of Breg. Similarly, an increase in transitional CD19^+^CD38^+^CD24^+^IgD^+^ B cells capable of producing IL-10 was found in long-term drug free KTRs (6).

Interestingly, it has previously been shown that regulatory T cells (Treg) levels are transiently increased after alemtuzumab induction (24), especially when calcineurin inhibitors are avoided (12,25). Moreover, immunosenescent CD8^+^CD28^−^ cells capable of suppressing CD4^+^ T-cell proliferation homeostatically proliferate (26). It therefore appears that alemtuzumab induction therapy creates an environment in which various cells with regulatory properties may act in concert.

In a rat model of long-term kidney transplantation tolerance, a shift in both peripheral and intragraft gene expression from IgG to IgM was observed, as well as IgM^+^, but not IgG^+^ B-cell clusters within the graft (27). In line with these data, polyclonally stimulated B cells from alemtuzumab-treated KTRs showed unaltered capacity to produce IgM, whereas we observed a dramatic decrease in IgG levels and number of IgG producing cells. This was not solely due to the absence of T cells in the circulation, as the attenuated IgG response was present at least up to 12 months after transplantation, at which time T cells were repopulated to 63% of the pretransplant level (Figure 1). These functional data confirm the long-term phenotypic arrest of peripheral B cells in a naïve state after alemtuzumab therapy. The fact that there is no increase in the number of IgM producing cells suggests that a proportion of B cells that repopulate after alemtuzumab induction are nonresponsive.

Our observation that the B-cell composition at 12 months in KTRs on standard triple therapy is similar to those who have undergone conversion to sirolimus suggests that alemtuzumab is the major driver of the long-term change of B-cell phenotype rather than the maintenance immunosuppressive regimen. Of note, basiliximab induction therapy did not affect the B-cell subset distribution (Figure S2). It is known from *in vitro* studies that both MMF and sirolimus are potent inhibitors of B-cell activation, whereas calcineurin inhibitors mainly inhibit B-cell activation through the inhibition of T-cell help (16,28). Whether sirolimus and calcineurin inhibitors differentially affect B-cell repopulation after induction therapy has not yet been investigated systematically, although our data suggest that this is not the case.

In several studies on autoimmune mediated diseases in which B cells were depleted with rituximab, memory B-cell repopulation after treatment correlated with relapse and worse clinical outcome (29,30). Similar observations have been made in kidney transplantation, where rituximab has been used to treat CD20^+^ acute rejection (23). Graft loss was associated with a shift from naïve toward memory B cells. In our study population, we found a very homogeneous B-cell depletion and none of the patients experienced a rejection episode during the study period. It will be interesting to study a larger cohort of patients to determine whether high levels of memory B cells after therapy correlate with worse transplant outcome.

In a mouse model of B-cell repopulation, the generation of a new B-cell repertoire in the presence of alloantigen resulted in humoral transplantation tolerance by elimination of alloantigen specific B cells in the transitional phase (31). Clearly in alemtuzumab-treated KTRs, B-cell repopulation occurs in the presence of alloantigen in the form of the allograft. It is tempting to speculate that long-term surviving alemtuzumab-treated KTRs may exhibit some degree of specific immunological unresponsiveness to donor alloantigens, particularly when taken alongside that this phenotype has also been found as part of the tolerance signature in immunosuppression free KTRs (5,6) and that alemtuzumab induction therapy may allow reduced immunosuppression in the longer term (13,32–34). In support of this conclusion, studies on the T-cell compartment suggest that Treg are present in alemtuzumab-treated KTRs treated with sirolimus as maintenance immunosuppression (12,24,35) and that such Treg have the capacity to control Th17 cells (36).

However, before drawing such a conclusion and embarking upon immunosuppression withdrawal studies, we believe it is critical that additional studies to address the function of the immune system are performed, not least because B-cell activating factor (BAFF) has been reported to be elevated in alemtuzumab-treated KTRs (37). Because BAFF plays an important role in breaking B-cell tolerance by providing survival signals to transitional B cells (38,39), increased BAFF levels have the potential to lower the threshold for the development of autoreactive, and in this case, alloreactive B cells. Indeed, when sirolimus was used as maintenance immunosuppression directly after alemtuzumab induction a high incidence of antibody-mediated rejection (AMR) was reported (11,25,40). Importantly in the KTRs studied here, we found no evidence for increased humoral alloreactivity, because none of the patients developed AMR. This observation may be due to the inclusion of a calcineurin inhibitor as maintenance immunosuppressive therapy (25).

In conclusion, data presented here show that in KTRs treated with alemtuzumab induction therapy, B cells with characteristics associated with operational tolerance reconstitute the immune system, suggesting that lymphocyte depletion with alemtuzumab may, at least partially, create an environment in which tolerance may be achieved. Additional work needs to be performed to determine the role of the B-cell subset distribution on long-term transplant outcome.

## Abbreviations

AMR: antibody-mediated rejection
ATG: anti-thymocyte globulin
BAFF: B cell activating factor
Breg: regulatory B cells
CPM: counts per minute
ELISPOT: Enzyme-linked immunosorbent spot assay
FCM: flow cytometric analysis
FCS: fetal calf serum
Ig: immunoglobulin
IL: interleukin
IMDM: Iscove's Modified Dulbecco's Medium
ITS: insulin, transferrin, selenium
KTRs: kidney transplant recipients
MMF: mycophenylate mofetil
MS: multiple sclerosis
PBMC: peripheral blood mononuclear cells
SFC: spot-forming cell
Treg: regulatory T cells

## Supporting information

**Figure S1: The total number of B cells, as well as absolute numbers of B cells of the transitional and naïve B-cellsubsets are increased at 6 months after alemtuzumab treatment, whereas absolute number of memory B cells are decreased.** Absolute number of B-cell subsets were calculated using percentages of B-cell subsets from flow cytometric analysis and absolute lymphocyte counts obtained from clinical laboratory reports.

**Figure S2: B-cell phenotype after basiliximab induction therapy up to 12 months after transplantation.**
