# Supplementary material for: B Cell Repopulation After Alemtuzumab Induction—Transient Increase in Transitional B Cells and Long-Term Dominance of Naïve B Cells
Source: Am J Transplant. 2012 Jul;12(7):1784–92. doi: 10.1111/j.1600-6143.2012.04012.x (PMC3387484; doi:10.1111/j.1600-6143.2012.04012.x)
Supplement: Supplementary file 1 [file ajt0012-1784-SD1.docx]

B Cell repopulation after Alemtuzumab induction – TRaNsient Increase in TRANSITIONAL b cells and LONG TERM DOminance of naïve B cells

Sebastiaan Heidt^1^, Joanna Hester^1^, Sushma Shankar^1^, Peter J. Friend^2^ and Kathryn J. Wood^1^

^1^Transplant Research Immunology Group, Nuffield Department of Surgical Sciences, University of Oxford, Oxford, United Kingdom

^2^Oxford Transplant Centre, Nuffield Department of Surgical Sciences, University of Oxford, Oxford, United Kingdom

*Corresponding author*

Kathryn J. Wood

Nuffield Department of Surgical Sciences, University of Oxford

Level 6, John Radcliffe Hospital, Oxford, OX3 9DU

kathryn.wood@nds.ox.ac.uk

*Running title*: B cell repopulation after alemtuzumab therapy

*Keywords*: Campath-1H, depletion, renal transplant, regulatory B cells, conversion

*Word count*

Abstract: 195, main body: 3191

Tables: 1, figures: 4, supplementary figures: 2

*Funding*

This study was supported by grants from the Roche Organ Transplant Research Foundation and The Wellcome Trust.

**Abbreviations**

AMR: antibody-mediated rejection

ATG: anti-thymocyte globulin

BAFF: B cell activating factor

Breg: regulatory B cells

CPM: counts per minute

ELISPOT: Enzyme-linked immunosorbent spot assay

FCM: flow cytometric analysis

FCS: fetal calf serum

Ig: immunoglobulin

IL: interleukin

IMDM: Iscove’s Modified Dulbecco’s Medium

ITS: insulin, transferrin, selenium

KTRs: kidney transplant recipients

MMF: mycophenylate mofetil

MS: multiple sclerosis

PBMC: peripheral blood mononuclear cells

SFC: spot-forming cell

Treg: regulatory T cells

**Abstract**

In organ transplantation, the composition of the B cell compartment is increasingly identified as an important determinant for graft outcome. Whereas naïve and transitional B cells have been associated with long-term allograft survival and operational tolerance, memory B cells have been linked to decreased allograft survival. Alemtuzumab induction therapy effectively depletes B cells, but is followed by rapid repopulation up to levels exceeding base line. The characteristics of the repopulating B cells are currently unknown. We studied the phenotypic and functional characteristics of B cells longitudinally in 19 kidney transplant recipients, before and at 6, 9 and 12 months after alemtuzumab induction therapy. A transient increase in transitional B cells and cells with phenotypic characteristics of regulatory B cells, as well as a long-term dominance in naïve B cells was found in alemtuzumab treated kidney transplant recipients, which was not influenced by conversion from tacrolimus to sirolimus. At all time-points after treatment, B cells showed unaltered proliferative and IgM-producing capacity as compared to pre-transplant samples, whereas the ability to produce IgG was inhibited long-term. In conclusion, induction therapy with alemtuzumab results in a long-term shift towards naïve B cells with altered phenotypic and functional characteristics.

**Introduction**

Humoral immunity is increasingly recognized as an important component of the alloimmune response. It is clear that alloantibodies produced by B cells can cause (hyper)acute graft rejection and it has been postulated that production of donor specific alloantibodies after transplantation may be an important cause of late graft loss ([1](#_ENREF_1), [2](#_ENREF_2)). Interestingly, and in contrast, novel data suggest that B cells may play an important role in allograft survival and the development of operational transplant tolerance (reviewed in ([3](#_ENREF_3))).

Recently, a B cell signature of tolerance has been described in immunosuppressive drug free long-term surviving kidney transplant recipients (KTRs), including an increase in the total number of peripheral B cells ([4](#_ENREF_4)) and a relative increase in naïve and transitional B cells, when compared to KTRs with stable graft function or biopsy proven chronic rejection ([5](#_ENREF_5), [6](#_ENREF_6)). A distinct, but also B cell dominated signature of tolerance was identified in a separate cohort of long-term immunosuppression free KTRs ([7](#_ENREF_7)). Taken together, these data suggest a potential role for B cells in the development and/or maintenance of operational tolerance in KTRs ([8](#_ENREF_8)).

Characterisation of the immune phenotype of patients with surviving allografts in the absence of immunosuppressive drugs compliments experimental studies exploring novel strategies to actively induce transplantation tolerance. In a nonhuman primate model of islet transplantation, long-term allograft survival achieved by T and B cell depletion with anti-thymocyte globulin (ATG) and rituximab was associated with the emergence and persistence of immature and transitional B cells ([9](#_ENREF_9)). These data suggest that therapies that drive the B cell compartment towards a ‘tolerant’ phenotype could potentially aid in the establishment of transplantation tolerance.

The anti-CD52 specific humanized monoclonal antibody alemtuzumab rapidly depletes T cells, B cells, NK cells and monocytes from the circulation, and is increasingly being used as induction therapy for kidney transplantation ([10](#_ENREF_10)). After depletion, monocytes rapidly repopulate, followed by NK cells and B cells ([11-13](#_ENREF_11)). Strikingly, while T cells can take years to repopulate to pre-treatment levels, B cells often repopulate up to levels exceeding pre-treatment levels within a year after treatment ([12](#_ENREF_12), [14](#_ENREF_14)).

Although repopulating B cells of patients with multiple sclerosis (MS) treated with alemtuzumab have been shown to be mainly naïve ([14](#_ENREF_14)), information about the composition of the repopulating B cell pool and the influence of maintenance immunosuppressive therapy in KTRs after alemtuzumab induction is scarce ([15](#_ENREF_15)). In the light of the increasingly appreciated role of B cells in transplant rejection and tolerance, characterisation of repopulating B cells after alemtuzumab induction is of great interest. We therefore hypothesised that repopulating B cells in alemtuzumab treated KTRs would show an altered phenotypic and functional profile compared to that pre-transplant and that this would not be impacted by post-transplant immunosuppression.

**Materials and methods**

*Patients*

KTRs treated with alemtuzumab induction therapy (two doses of 30 mg i.v.) were included (n=19). Fifteen patients received maintenance immunosuppressive therapy consisting of tacrolimus (target trough level 5-8 ng/mL), MMF (500 mg, twice daily) and steroids. Four patients did not receive steroids and were converted from tacrolimus to low dose sirolimus (adjusted to 5-8 ng/mL) at 6 months, followed by MMF withdrawal at 12 months. The study was approved by Oxfordshire Research Ethics Committee B under the reference numbers 07/H0603/42 and C02.225. Patients were recruited after informed consent and blood was taken before and at several time points up to 12 months after transplantation.

*Cells*

Peripheral blood mononuclear cells (PBMC) were isolated by Ficoll-Paque (GE Healthcare, Uppsala, Sweden) gradient centrifugation and stored in liquid nitrogen until further use. B cells were immunomagnetically isolated using Dynabeads CD19 pan B and Detach-a-Bead CD19 (Invitrogen, San Diego, CA). Cell cultures were performed in culture medium consisting of Iscove’s modified Dulbecco’s medium (IMDM) supplemented with 10% fetal calf serum (FCS), 100 U/mL penicillin, 100 μg/mL streptomycin (all from PAA Laboratories, Pasching, Austria), 0.05 mM 2-mercaptoethanol (Sigma-Aldrich, St. Louis, MO), and ITS (insulin 5 μg/mL, transferrin 5 μg/mL, and selenium 5 ng/ml, Sigma-Aldrich).

*Flow cytometry*

FCM was performed according to standard protocols using the following antibodies (clone): CD19 (SJ25C1), CD27 (M-T271), CD24 (ML5), CD5 (UCHT2), IgD (IA6-2), IgM (G20-127) (all from BD Biosciences, Oxford, UK), CD10 (ALB1) (Beckman Coulter, Fullerton, CA), CD20 (2H7), CD38 (HIT2) (eBioscience, San Diego, CA), CD1d (51.1) (Biolegend San Diego, CA) or relevant isotype controls.

*B cell activation*

B cells were cultured at 1x10^5^ cells/well in 96-well roundbottom plates (Corning, Amsterdam, the Netherlands) and activated with 500 ng/mL of agonistic anti-CD40, 25 ng/mL of interleukin (IL)-10 (both from R&D systems, Abingdon, UK), 100 ng/mL of IL-21 (Invitrogen), 100 U/mL of IL-2 (Chiron, Emeryville, CA) and 2.5 μg/mL of ODN-2006 CpG (Hycult Biotechnology, Uden, the Netherlands).

*Proliferation assay*

B cells were activated as described above for 7 d. At day 6, supernatants were collected for Ig detection and 1 μCi ^3^H-TdR (Perkin Elmer, Cambridge, UK) was added per well for the last 16 h of culture. ^3^H-TdR incorporation was measured using a liquid scintillation counter (Wallac, Turku, Finland).

*Immunoglobulin Production*

ELISA and ELISPOT assays to quantify IgM and IgG levels in culture supernatants and the number of B cells producing IgM and IgG were performed as described previously ([16](#_ENREF_16)).

*Statistics*

The repeated measures ANOVA with post testing by Dunnett Multiple Comparisons Test was used for comparisons of variables in time, whereas the unpaired T test was used to analyse differences between treatment groups; *P*-values <0.05 were considered significant. Results in the text are expressed as mean ± sd.

**Results**

*Repopulating B cells after alemtuzumab induction show a naïve phenotype*

After alemtuzumab-induced leukocyte depletion, B cells repopulated the peripheral blood of KTRs from 6 weeks onwards and exceeded base line levels from 6 months (Figure 1). T cells started to repopulate after 3 months, not reaching base line levels within the one-year time frame, confirming previous reports ([11](#_ENREF_11), [12](#_ENREF_12)).

B cell differentiation stages can be identified by flow cytometric analysis (FCM) using various classification schemes, of which the CD27-IgD ([17](#_ENREF_17)) and the Bm1-Bm5 classification ([18](#_ENREF_18)) are most commonly used. Using the CD27-IgD classification scheme (Figure 2A), we observed that following alemtuzumab treatment, there was a clear shift to lower levels of memory B cells in the peripheral blood; 19.1 ± 7.0% memory B cells (including IgD^+^CD27^+^ non-switched memory B cells and IgD^-^CD27^+^ switched memory B cells) pre-induction compared to 2.0 ± 1.6% at 6 months post-transplant (p<0.01). The proportion of memory B cells remained low in alemtuzumab treated KTRs for 12 months after treatment (2.4% ± 1.9, p<0.01, Figure 2B). The IgD^-^CD27^-^ B cell population, which has been described as comprising exhausted memory B cells ([19](#_ENREF_19)), was also decreased from 7.9 ± 6.9% pre-induction to 1.9 ± 1.2% at 6 months (p<0.01), remaining low up to 12 months (2.3 ± 1.4%, p<0.05, Figure 2C). Consequently, the naive B cell compartment (IgD^+^CD27^-^) was highly enriched from a mean of 73.6 ± 8.3% pre-treatment to 96.7 ± 2.1% at 6 months after treatment (p<0.01), and remained high at 12 months after transplantation (95.6 ± 2.8%, p<0.01, Figure 2D).

When using IgD and CD38 to identify B cells into Bm1-Bm5 subsets (Figure 2E), we observed a decrease in Bm1 cells from 9.3% ± 3.8 pre-treatment to 2.0 ± 1.4% at 6 months (p<0.01) that remained attenuated up to 12 months (5.0 ± 3.5%, p<0.01, Figure 2F). This was mainly due to the depletion of memory B cells that had not undergone class switching (non-switched memory B cells) that reside in this gate besides ‘virgin naïve’ B cells, as determined by CD27 positivity (data not shown). Bm2 cells (comprising activated naïve B cells) were increased from 67.2 ± 9.2% before treatment to 78.7 ± 6.3% at 6 months (p<0.01). This subpopulation continued to rise at 12 months after treatment, up to 85.3 ± 4.1% (p<0.01, Figure 2G).

Cells in the Bm2’ gate were transiently increased from 4.3 ± 3.5% pre-treatment to 18.0 ± 6.7% at 6 months (p<0.01), after which levels dropped near to baseline levels (6.7 ± 1.9%, p=ns, Figure 2H). Since these cells were mainly CD27^-^, they have the phenotypic characteristics of transitional B cells, rather than pre-germinal B cells, which are CD27^+^ ([20](#_ENREF_20), [21](#_ENREF_21)). Recently, a population of human regulatory B cells (Breg) residing within the transitional B cell compartment expressing high levels of CD24 and CD38 has been described ([22](#_ENREF_22)). In alemtuzumab treated KTRs, we observed a transient increase in peripheral B cells expressing high levels of CD24 and CD38 at 6 months after transplantation (Figure 3A; 2.2 ± 2.0% pre-treatment vs 8.9 ± 3.9% at 6 months (p<0.01)). These cells had the phenotypic characteristics of human Breg ([22](#_ENREF_22)), since these cells were IgM^hi^IgD^hi^CD5^+^CD10^+^CD20^+^CD27^-^CD1d^+^ (Figure 3C). Analysis at later time points demonstrated that this subpopulation then declined to lower levels; 4.5 ± 2.1% at 9 months (p=ns) and 3.1 ± 1.5% at 12 months (p=ns, Figure 3B).

The germinal centre Bm3+4 subsets were virtually absent (Figure 2I), as has been described previously for peripheral blood ([18](#_ENREF_18)). Consistent with results from the IgD-CD27 classification system, the percentage of both early and late memory cells (Bm5) were profoundly decreased after alemtuzumab induction. Early Bm5 cells decreased from 10.1 ± 4.3% to 1.6 ± 1.0% at 6 months (p<0.01), remaining low at 12 months (1.9 ± 1.3%, p<0.01, Figure 2J), whereas late Bm5 cells decreased from 8.9 ± 4.6% to 0.7 ± 0.5% at 6 months (p<0.01), remaining low at 12 months (1.4 ± 1.1%, p<0.01, Figure 2K).

For 5 KTRs, we had the opportunity to determine the absolute number of B cells in each subset in fresh blood samples pre-transplant and at 6 months after transplantation. The observed changes in the absolute number of cells in each B cell subset confirmed our findings analysing stored frozen PBMC, indicating that in the peripheral blood of KTRs after alemtuzumab induction therapy the composition of B cells subsets is altered compared to that found pre-transplant; naïve and transitional B cells are increased, whereas the absolute number of memory B cells is significantly decreased (Figure S1).

*Repopulating B cells have an altered response to polyclonal stimulation*

To investigate the functional properties of repopulating B cells from KTRs treated with alemtuzumab induction therapy, B cells were stimulated polyclonally and found to proliferate with the same extent to that of B cells before alemtuzumab induction (Figure 4A, p=ns for any time point). However, whereas similar amounts of IgM were detected in the supernatant of the B cell cultures both pre- and post-induction (p=ns for any time point), the amount of IgG detected was profoundly decreased from 9.8 ± 6.0 μg/ml in pre-transplant B cell cultures to 1.1 ± 1.0 μg/ml at 6 months (p<0.01), 1.3 ± 1.0 μg/ml at 9 months (p<0.01) and 1.8 ± 1.0 μg/ml at 12 months after treatment (p<0.01, Figure 4B).

To verify whether this was due to a reduction of the number of cells producing IgG, we performed Ig ELISPOT assays on polyclonally stimulated B cells. Compared to pre-treatment cultures, the number of B cells producing IgM was unaltered (p=ns for any time point). In contrast, the number of B cells producing IgG after polyclonal stimulation was dramatically decreased from 186 ± 52 spot-forming cells (SFC) per 1000 B cells pre-treatment to 54 ± 15 SFC at 6 months (p<0.01), 56 ± 12 SFC at 9 months (p<0.01) and 60 ± 19 SFC at 12 months post-treatment (p<0.01, Figure 4C).

*Long-term immunosuppression does not influence B cell repopulation*

To establish whether maintenance immunosuppression would influence the distribution of B cell subsets, we determined the phenotype and composition of B cells in the peripheral blood of alemtuzumab treated KTRs who were enrolled in a conversion trial in which immunosuppression was converted from tacrolimus to low dose sirolimus at 6 months after transplantation. B cell subsets at 12 months in these patients were compared to those in alemtuzumab treated KTRs who remained on standard triple therapy. As shown in Table 1, we did not find any differences in B cell subsets between patients converted to sirolimus and patients that remained on standard triple therapy.

**Discussion**

Increasing evidence suggests that B cells may contribute to transplant tolerance, potentially resulting in successful long-term graft outcome. In this study, we have shown that after alemtuzumab induction, the repopulating B cell compartment is altered compared to that pre-transplant, comprising mainly naïve B cells that produce IgM upon activation, and that these remain the dominant cell type for at least 12 months after alemtuzumab therapy. Furthermore, we have shown that following alemtuzumab treatment there are dynamic changes in the repopulating B cell pool with a transient increase in transitional B cells, including B cells with phenotypic characteristics of Breg.

The composition of the B cell compartment has been identified as an important factor for graft outcome. An increase in the number of naïve B cells and percentage of transitional B cells has been described in long-term immunosuppressive drug free tolerant KTRs, suggesting that a shift towards a naïve/transitional B cell phenotype might be a prerequisite for the development of tolerance ([6](#_ENREF_6)). Similarly, in a nonhuman primate model of islet transplantation, B cell reconstitution after rituximab was dominated by immature and transitional B cells whose persistence was associated with long-term insulin independence ([9](#_ENREF_9)). Moreover, in KTRs treated with rituximab for CD20^+^ acute rejection, a decrease in naïve B-cells was associated with graft loss ([23](#_ENREF_23)).

After alemtuzumab induction, we observed a shift towards naïve B cells in peripheral blood, as determined by different B cell classification schemes. Consequently, B cells with a memory phenotype were virtually absent for at least 12 months after induction therapy. We also showed that alemtuzumab treatment resulted in a transient increase in cells that have phenotypic characteristics of Breg. Similarly, an increase in transitional CD19^+^CD38^+^CD24^+^IgD^+^ B cells capable of producing IL-10 was found in long-term drug free KTRs ([6](#_ENREF_6)).

Interestingly, it has previously been shown that regulatory T cells (Treg) levels are transiently increased after alemtuzumab induction ([24](#_ENREF_24)), especially when calcineurin inhibitors are avoided ([12](#_ENREF_12), [25](#_ENREF_25)). Moreover, immunosenescent CD8^+^CD28^-^ cells capable of suppressing CD4^+^ T cell proliferation homeostatically proliferate ([26](#_ENREF_26)). It therefore appears that alemtuzumab induction therapy creates an environment in which various cells with regulatory properties may act in concert.

In a rat model of long-term kidney transplantation tolerance, a shift in both peripheral and intragraft gene expression from IgG to IgM was observed, as well as IgM^+^, but not IgG^+^ B cell clusters within the graft ([27](#_ENREF_27)). In line with these data, polyclonally stimulated B cells from alemtuzumab treated KTRs showed unaltered capacity to produce IgM, whereas we observed a dramatic decrease in IgG levels and number of IgG producing cells. This was not solely due to the absence of T cells in the circulation, as the attenuated IgG response was present at least up to 12 months after transplantation, at which T cells were repopulated to 63% of the pre-transplant level (Figure 1). These functional data confirm the long-term phenotypic arrest of peripheral B cells in a naïve state after alemtuzumab therapy. The fact that there is no increase in the number of IgM producing cells suggests that a proportion of B cells that repopulate after alemtuzumab induction are non-responsive.

Our observation that the B cell composition at 12 months in KTRs on standard triple therapy is similar to those who have undergone conversion to sirolimus suggests that alemtuzumab is the major driver of the long-term change of B cell phenotype rather than the maintenance immunosuppressive regimen. Of note, basiliximab induction therapy did not affect the B cell subset distribution (Figure S2). It is known from *in vitro* studies that both MMF and sirolimus are potent inhibitors of B cell activation, whereas calcineurin inhibitors mainly inhibit B cell activation through the inhibition of T cell help ([16](#_ENREF_16), [28](#_ENREF_28)). Whether sirolimus and calcineurin inhibitors differentially affect B cell repopulation after induction therapy has not yet been investigated systematically, although our data suggest that this is not the case.

In several studies on autoimmune mediated diseases in which B cells were depleted with rituximab, memory B cell repopulation after treatment correlated with relapse and worse clinical outcome ([29](#_ENREF_29), [30](#_ENREF_30)). Similar observations have been made in kidney transplantation, where rituximab has been used to treat CD20^+^ acute rejection ([23](#_ENREF_23)). Graft loss was associated with a shift from naïve towards memory B cells. In our study population, we found a very homogeneous B cell depletion and none of the patients experienced a rejection episode during the study period. It will be interesting to study a larger cohort of patients to determine whether high levels of memory B cells after therapy correlate with worse transplant outcome.

In a mouse model of B cell repopulation, the generation of a new B cell repertoire in the presence of alloantigen resulted in humoral transplantation tolerance by elimination of alloantigen specific B cells in the transitional phase ([31](#_ENREF_31)). Clearly in alemtuzumab treated KTRs, B cell repopulation occurs in the presence of alloantigen in the form of the allograft. It is tempting to speculate that long term surviving alemtuzumab treated KTRs may exhibit some degree of specific immunological unresponsiveness to donor alloantigens, particularly when taken alongside that this phenotype has also been found as part of the tolerance signature in immunosuppression free KTRs ([5](#_ENREF_5), [6](#_ENREF_6)) and that alemtuzumab induction therapy may allow reduced immunosuppression in the longer term ([13](#_ENREF_13), [32-34](#_ENREF_32)). In support of this conclusion, studies on the T cell compartment suggest that Treg are present in alemtuzumab treated KTRs treated with sirolimus as maintenance immunosuppression ([12](#_ENREF_12), [24](#_ENREF_24), [35](#_ENREF_35)) and that such Treg have the capacity to control Th17 cells ([36](#_ENREF_36)).

However, before drawing such a conclusion and embarking upon immunosuppression withdrawal studies, we believe it is critical that additional studies to address the function of the immune system are performed, not least because B cell activating factor (BAFF) has been reported to be elevated in alemtuzumab treated KTRs ([37](#_ENREF_37)). Since BAFF plays an important role in breaking B cell tolerance by providing survival signals to transitional B cells ([38](#_ENREF_38), [39](#_ENREF_39)), increased BAFF levels have the potential to lower the threshold for the development of autoreactive, and in this case, alloreactive B cells. Indeed, when sirolimus was used as maintenance immunosuppression directly after alemtuzumab induction a high incidence of antibody-mediated rejection (AMR) was reported ([11](#_ENREF_11), [25](#_ENREF_25), [40](#_ENREF_40)). Importantly in the KTRs studied here, we found no evidence for increased humoral alloreactivity, since none of the patients developed AMR. This observation may be due to the inclusion of a calcineurin inhibitor as maintenance immunosuppressive therapy ([25](#_ENREF_25)).

In conclusion, data presented here show that in KTRs treated with alemtuzumab induction therapy, B cells with characteristics associated with operational tolerance reconstitute the immune system, suggesting that lymphocyte depletion with alemtuzumab may, at least partially, create an environment in which tolerance may be achieved. Additional work needs to be performed to determine the role of the B cell subset distribution on long-term transplant outcome.

**Acknowledgements**

This study was supported by grants from the Roche Organ Transplant Research Foundation and The Wellcome Trust. The authors thank David San Segundo for sample processing and Sally Ruse and staff of the Oxford Transplant Centre for help with sample acquisition.

**Disclosures**

The authors of this manuscript have no conflicts of interest to disclose as described by the American Journal of Transplantation.

**References**

1. Terasaki PI, Cai J. Human leukocyte antigen antibodies and chronic rejection: from association to causation. Transplantation 2008;86(3):377-383.

2. Wood KJ, Goto R. Mechanisms of rejection: current perspectives. Transplantation 2012;93(1):1-10.

3. Kirk AD, Turgeon NA, Iwakoshi NN. B cells and transplantation tolerance. Nat Rev Nephrol 2010.

4. Louis S, Braudeau C, Giral M, Dupont A, Moizant F, Robillard N et al. Contrasting CD25hiCD4+T cells/FOXP3 patterns in chronic rejection and operational drug-free tolerance. Transplantation 2006;81(3):398-407.

5. Sagoo P, Perucha E, Sawitzki B, Tomiuk S, Stephens DA, Miqueu P et al. Development of a cross-platform biomarker signature to detect renal transplant tolerance in humans. J Clin Invest 2010;120(6):1848-1861.

6. Newell KA, Asare A, Kirk AD, Gisler TD, Bourcier K, Suthanthiran M et al. Identification of a B cell signature associated with renal transplant tolerance in humans. J Clin Invest 2010;120(6):1836-1847.

7. Pallier A, Hillion S, Danger R, Giral M, Racape M, Degauque N et al. Patients with drug-free long-term graft function display increased numbers of peripheral B cells with a memory and inhibitory phenotype. Kidney Int 2010;78(5):503-513.

8. Heidt S, San Segundo D, Shankar S, Mittal S, Muthusamy AS, Friend PJ et al. Peripheral blood sampling for the detection of allograft rejection: biomarker identification and validation. Transplantation 2011;92(1):1-9.

9. Liu C, Noorchashm H, Sutter JA, Naji M, Prak EL, Boyer J et al. B lymphocyte-directed immunotherapy promotes long-term islet allograft survival in nonhuman primates. Nature medicine 2007;13(11):1295-1298.

10. Markmann JF, Fishman JA. Alemtuzumab in kidney-transplant recipients. The New England journal of medicine 2011;364(20):1968-1969.

11. Knechtle SJ, Pirsch JD, H. Fechner J J, Becker BN, Friedl A, Colvin RB et al. Campath-1H induction plus rapamycin monotherapy for renal transplantation: results of a pilot study. Am J Transplant 2003;3(6):722-730.

12. Noris M, Casiraghi F, Todeschini M, Cravedi P, Cugini D, Monteferrante G et al. Regulatory T cells and T cell depletion: role of immunosuppressive drugs. J Am Soc Nephrol 2007;18(3):1007-1018.

13. Trzonkowski P, Zilvetti M, Friend P, Wood KJ. Recipient memory-like lymphocytes remain unresponsive to graft antigens after CAMPATH-1H induction with reduced maintenance immunosuppression. Transplantation 2006;82(10):1342-1351.

14. Thompson SA, Jones JL, Cox AL, Compston DA, Coles AJ. B-cell reconstitution and BAFF after alemtuzumab (Campath-1H) treatment of multiple sclerosis. J Clin Immunol 2010;30(1):99-105.

15. Knechtle SJ, Pascual J, Bloom DD, Torrealba JR, Jankowska-Gan E, Burlingham WJ et al. Early and limited use of tacrolimus to avoid rejection in an alemtuzumab and sirolimus regimen for kidney transplantation: clinical results and immune monitoring. Am J Transplant 2009;9(5):1087-1098.

16. Heidt S, Roelen DL, Eijsink C, van Kooten C, Claas FH, Mulder A. Effects of immunosuppressive drugs on purified human B cells: evidence supporting the use of MMF and rapamycin. Transplantation 2008;86(9):1292-1300.

17. Klein U, Rajewsky K, Kuppers R. Human immunoglobulin (Ig)M+IgD+ peripheral blood B cells expressing the CD27 cell surface antigen carry somatically mutated variable region genes: CD27 as a general marker for somatically mutated (memory) B cells. The Journal of experimental medicine 1998;188(9):1679-1689.

18. Bohnhorst JO, Bjorgan MB, Thoen JE, Natvig JB, Thompson KM. Bm1-Bm5 classification of peripheral blood B cells reveals circulating germinal center founder cells in healthy individuals and disturbance in the B cell subpopulations in patients with primary Sjogren's syndrome. J Immunol 2001;167(7):3610-3618.

19. Colonna-Romano G, Bulati M, Aquino A, Pellicano M, Vitello S, Lio D et al. A double-negative (IgD-CD27-) B cell population is increased in the peripheral blood of elderly people. Mechanisms of ageing and development 2009;130(10):681-690.

20. Sims GP, Ettinger R, Shirota Y, Yarboro CH, Illei GG, Lipsky PE. Identification and characterization of circulating human transitional B cells. Blood 2005;105(11):4390-4398.

21. Sarantopoulos S, Stevenson KE, Kim HT, Washel WS, Bhuiya NS, Cutler CS et al. Recovery of B-cell homeostasis after rituximab in chronic graft-versus-host disease. Blood 2011;117(7):2275-2283.

22. Blair PA, Norena LY, Flores-Borja F, Rawlings DJ, Isenberg DA, Ehrenstein MR et al. CD19(+)CD24(hi)CD38(hi) B cells exhibit regulatory capacity in healthy individuals but are functionally impaired in systemic Lupus Erythematosus patients. Immunity 2010;32(1):129-140.

23. Zarkhin V, Lovelace PA, Li L, Hsieh SC, Sarwal MM. Phenotypic evaluation of B-cell subsets after rituximab for treatment of acute renal allograft rejection in pediatric recipients. Transplantation 2011;91(9):1010-1018.

24. Bloom DD, Chang Z, Fechner JH, Dar W, Polster SP, Pascual J et al. CD4+ CD25+ FOXP3+ regulatory T cells increase de novo in kidney transplant patients after immunodepletion with Campath-1H. Am J Transplant 2008;8(4):793-802.

25. Pascual J, Bloom D, Torrealba J, Brahmbhatt R, Chang Z, Sollinger HW et al. Calcineurin inhibitor withdrawal after renal transplantation with alemtuzumab: clinical outcomes and effect on T-regulatory cells. Am J Transplant 2008;8(7):1529-1536.

26. Trzonkowski P, Zilvetti M, Chapman S, Wieckiewicz J, Sutherland A, Friend P et al. Homeostatic repopulation by CD28-CD8+ T cells in alemtuzumab-depleted kidney transplant recipients treated with reduced immunosuppression. Am J Transplant 2008;8(2):338-347.

27. Le Texier L, Thebault P, Lavault A, Usal C, Merieau E, Quillard T et al. Long-term allograft tolerance is characterized by the accumulation of B cells exhibiting an inhibited profile. Am J Transplant 2011;11(3):429-438.

28. Heidt S, Roelen DL, Eijsink C, Eikmans M, van Kooten C, Claas FH et al. Calcineurin inhibitors affect B cell antibody responses indirectly by interfering with T cell help. Clin Exp Immunol 2010;159(2):199-207.

29. Anolik JH, Barnard J, Owen T, Zheng B, Kemshetti S, Looney RJ et al. Delayed memory B cell recovery in peripheral blood and lymphoid tissue in systemic lupus erythematosus after B cell depletion therapy. Arthritis Rheum 2007;56(9):3044-3056.

30. Roll P, Dorner T, Tony HP. Anti-CD20 therapy in patients with rheumatoid arthritis: predictors of response and B cell subset regeneration after repeated treatment. Arthritis Rheum 2008;58(6):1566-1575.

31. Parsons RF, Vivek K, Rostami SY, Zekavat G, Ziaie SM, Luo Y et al. Acquisition of humoral transplantation tolerance upon de novo emergence of B lymphocytes. Journal of immunology 2011;186(1):614-620.

32. Calne R, Moffatt SD, Friend PJ, Jamieson NV, Bradley JA, Hale G et al. Campath IH allows low-dose cyclosporine monotherapy in 31 cadaveric renal allograft recipients. Transplantation 1999;68(10):1613-1616.

33. Kirk AD, Hale DA, Mannon RB, Kleiner DE, Hoffmann SC, Kampen RL et al. Results from a human renal allograft tolerance trial evaluating the humanized CD52-specific monoclonal antibody alemtuzumab (CAMPATH-1H). Transplantation 2003;76(1):120-129.

34. Watson CJ, Bradley JA, Friend PJ, Firth J, Taylor CJ, Bradley JR et al. Alemtuzumab (CAMPATH 1H) induction therapy in cadaveric kidney transplantation--efficacy and safety at five years. Am J Transplant 2005;5(6):1347-1353.

35. Ruggenenti P, Perico N, Gotti E, Cravedi P, D'Agati V, Gagliardini E et al. Sirolimus versus cyclosporine therapy increases circulating regulatory T cells, but does not protect renal transplant patients given alemtuzumab induction from chronic allograft injury. Transplantation 2007;84(8):956-964.

36. Hester J, Mills N, Shankar S, Carvalho-Gaspar M, Friend P, Wood KJ. Th17 cells in alemtuzumab-treated patients: the effect of long-term maintenance immunosuppressive therapy. Transplantation 2011;91(7):744-750.

37. Bloom D, Chang Z, Pauly K, Kwun J, Fechner J, Hayes C et al. BAFF is increased in renal transplant patients following treatment with alemtuzumab. Am J Transplant 2009;9(8):1835-1845.

38. Mackay F, Woodcock SA, Lawton P, Ambrose C, Baetscher M, Schneider P et al. Mice transgenic for BAFF develop lymphocytic disorders along with autoimmune manifestations. J Exp Med 1999;190(11):1697-1710.

39. Thien M, Phan TG, Gardam S, Amesbury M, Basten A, Mackay F et al. Excess BAFF rescues self-reactive B cells from peripheral deletion and allows them to enter forbidden follicular and marginal zone niches. Immunity 2004;20(6):785-798.

40. Cai J, Terasaki PI, Bloom DD, Torrealba JR, Friedl A, Sollinger HW et al. Correlation between human leukocyte antigen antibody production and serum creatinine in patients receiving sirolimus monotherapy after Campath-1H induction. Transplantation 2004;78(6):919-924.

Figure legends

**Figure 1.** (A) Absolute number of CD19^+^ B cells and (B) CD3^+^ T cells in peripheral blood of alemtuzumab treated KTRs up to 12 months after treatment (n=10). The absolute number of cells was determined by multiplying the respective percentages obtained by flow cytometry by absolute lymphocyte counts from clinical laboratory reports.The dotted lines represent pre-transplant values.

**Figure 2.** Phenotype of repopulating B cells after alemtuzumab induction therapy up to 12 months after transplantation. (A) Representative dot plots of repopulating B cells using the CD27-IgD classification. (B) Percentage of naïve B cells (IgD^+^CD27^-^) within CD19 gate, (C) percentage of memory B cells (both IgD^-^CD27^+^ and IgD^+^CD27^+^) within CD19 gate, and (D) percentage of exhausted memory B cells (IgD^-^CD27^-^) within CD19 gate (n=7). (E) Representative dot plots of repopulating B cells using the Bm1-Bm5 classification. (F) Percentage of Bm1 cells (IgD^+^CD38^-^) within CD19 gate, (G) percentage of Bm2 cells (IgD^+^CD38^int^) within CD19 gate, and (H) percentage of Bm2' cells (IgD^+^CD38^hi^) within CD19 gate, (I) percentage of Bm3+4 cells (IgD^-^CD38^hi^) within CD19 gate, (J) percentage of early Bm5 cells (IgD^-^CD38^int^) within CD19 gate, and (K) percentage of late Bm5 (IgD^-^CD38^-^) within CD19 gate (n=7). All statistics: repeated measures ANOVA with post testing by Dunnett Multiple Comparisons Test, *P<0.05, **P<0.01.

**Figure 3.** Transient increase of B cells with a Breg phenotype after alemtuzumab induction therapy. (A) Representative dot plots of CD19CD24^hi^CD38^hi^ B cells pre-transplantation and at 6 months after transplantation. (B) Percentage of CD24^hi^CD38^hi^ B cells within CD19 gate (n=7), statistics: repeated measures ANOVA with post testing by Dunnett Multiple Comparisons Test, **P<0.01. (C) CD24^hi^CD38^hi^ B cells have the phenotypic characteristics of Breg cells; IgM^hi^ and IgD^hi^, CD5^+^, CD10^+^, CD20^hi^, CD27^-^ and CD1d^+^.

**Figure 4.** Functional analysis of repopulating B cells after alemtuzumab induction therapy. (A) B cell proliferation after polyclonal stimulation pre-transplant and up to 12 months after alemtuzumab treatment analyzed by ^3^H-TdR incorporation (n=6). (B) IgM and IgG concentration in supernatants from polyclonally activated B cells pre-transplant and up to 12 months after alemtuzumab treatment analyzed by ELISA (n=5). (C) Results from IgM and IgG ELISPOT of polyclonally activated B cells pre-transplant and up to 12 months after alemtuzumab treatment (n=6). All statistics: repeated measures ANOVA with post testing by Dunnett Multiple Comparisons Test, **P<0.01.

**Supporting information**

Additional Supporting Information may be found in the online version of this article:

**Figure S1:** The total number of B cells, as well as absolute numbers of B cells of the transitional and naïve B cell subsets are increased at 6 months after alemtuzumab treatment, whereas absolute number of memory B cells are decreased. Absolute number of B cell subsets were calculated using percentages of B cell subsets from flow cytometric analysis and absolute lymphocyte counts obtained from clinical laboratory reports. Transitional B cells were defined as CD19^+^CD24^hi^CD38^hi^. Statistics: paired T test, *P<0.05, **P<0.01 (n=5).

**Figure S2:** B cell phenotype after basiliximab induction therapy up to 12 months after transplantation. Dot plots of kidney transplant recipient treated with basiliximab induction therapy followed by tacrolimus, MMF and steroids as maintenance immunosuppression are shown. (A) B cell phenotype according to the CD27-IgD classification and (B) according to the Bm1-Bm5 classification.

Table 1. Comparison of B cells subsets at 12 months after transplantation between patients converted to sirolimus at 6 months and patients on standard triple therapy

|  | B cells | Naïve | Memory | DN | Bm1 | Bm2 | Bm2’ | Bm3+4 | Early Bm5 | Late Bm5 | Transitional |
| --- | --- | --- | --- | --- | --- | --- | --- | --- | --- | --- | --- |
| No conversion (n=7) | 24.6 ± 19.8 | 95.6 ± 2.8 | 2.4 ± 1.9 | 2.3 ± 1.4 | 5.0 ± 3.5 | 85.3 ± 4.1 | 6.7 ± 1.9 | 0.4 ± 0.2 | 1.9 ± 1.3 | 1.4 ± 1.1 | 3.1 ± 1.5 |
| Conversion (n=4) | 26.6 ± 6.2 | 94.8 ± 3.9 | 2.9 ± 1.9 | 2.3 ± 2.1 | 3.8 ± 4.1 | 87.2 ± 8.2 | 4.7 ± 1.4 | 0.3 ± 0.1 | 1.9 ± 1.1 | 2.7 ± 2.9 | 3.0 ± 0.5 |
| P-value | 0.85 | 0.69 | 0.65 | 0.97 | 0.60 | 0.60 | 0.11 | 0.48 | 0.98 | 0.32 | 0.84 |

*Statistics: unpaired T test*
