# Supplementary figures and images for: B Cell Repopulation After Alemtuzumab Induction—Transient Increase in Transitional B Cells and Long-Term Dominance of Naïve B Cells
Source: Am J Transplant. 2012 Jul;12(7):1784–92. doi: 10.1111/j.1600-6143.2012.04012.x (PMC3387484; doi:10.1111/j.1600-6143.2012.04012.x)

Supplemental figure 1

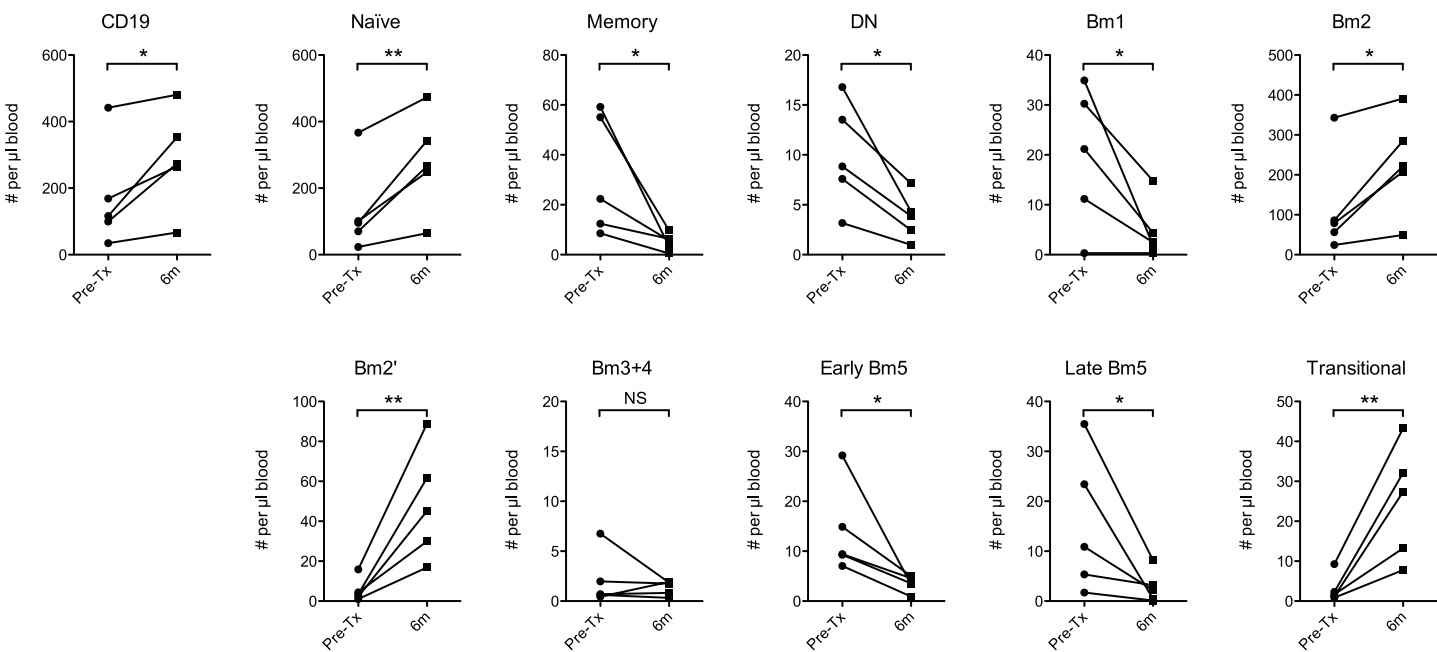

Supplement: Supplementary file 2 [file ajt0012-1784-SD2.pdf]

Supplemental figure 2

A

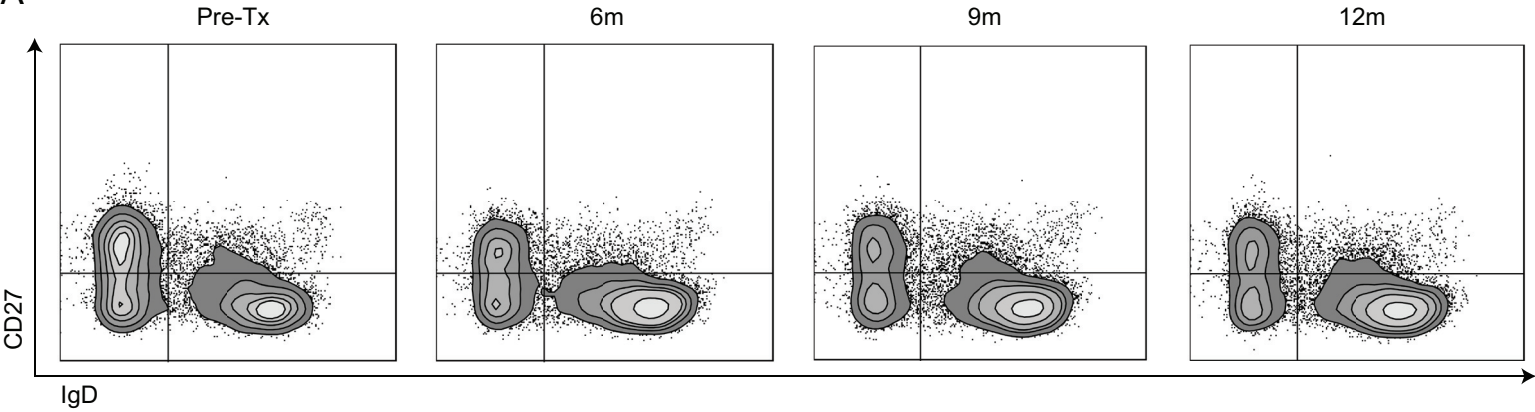

B

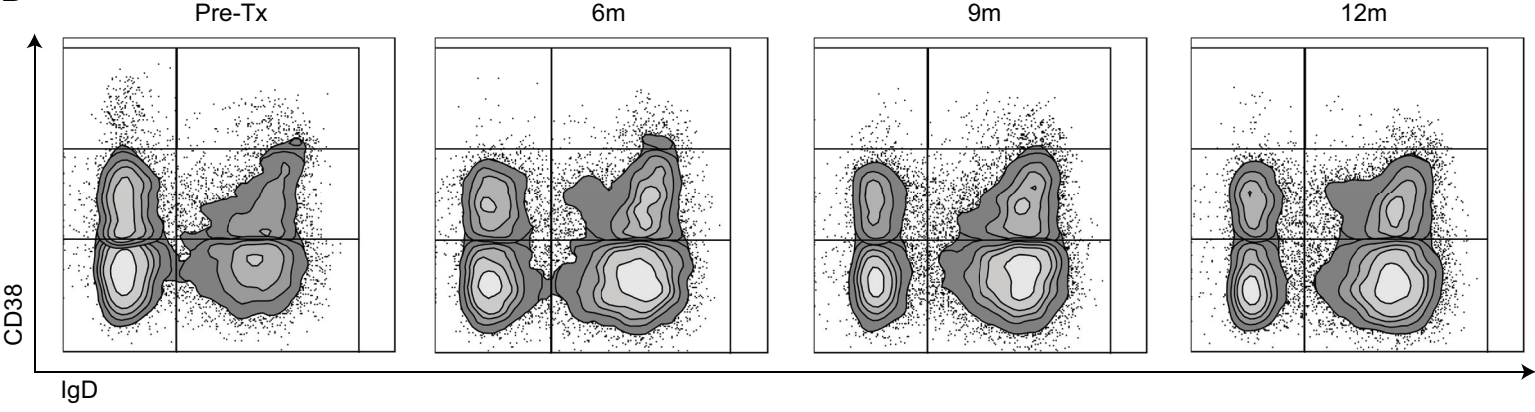

Supplement: Supplementary file 3 [file ajt0012-1784-SD3.pdf]
